# Thermodynamic Insights into Symmetry Breaking: Exploring Energy Dissipation across Diverse Scales

**DOI:** 10.3390/e26030231

**Published:** 2024-03-05

**Authors:** Andrés Arango-Restrepo, J. Miguel Rubi

**Affiliations:** Condensed Matter Department, Universitat de Barcelona, 08028 Barcelona, Spain; mrubi@ub.edu

**Keywords:** energy dissipation, entropy production, matter aggregation, mesostructures, self-assembly, self-organization, symmetry breaking

## Abstract

Symmetry breaking is a phenomenon that is observed in various contexts, from the early universe to complex organisms, and it is considered a key puzzle in understanding the emergence of life. The importance of this phenomenon is underscored by the prevalence of enantiomeric amino acids and proteins.The presence of enantiomeric amino acids and proteins highlights its critical role. However, the origin of symmetry breaking has yet to be comprehensively explained, particularly from an energetic standpoint. This article explores a novel approach by considering energy dissipation, specifically lost free energy, as a crucial factor in elucidating symmetry breaking. By conducting a comprehensive thermodynamic analysis applicable across scales, ranging from elementary particles to aggregated structures such as crystals, we present experimental evidence establishing a direct link between nonequilibrium free energy and energy dissipation during the formation of the structures. Results emphasize the pivotal role of energy dissipation, not only as an outcome but as the trigger for symmetry breaking. This insight suggests that understanding the origins of complex systems, from cells to living beings and the universe itself, requires a lens focused on nonequilibrium processes

## 1. Introduction

Symmetry breaking stands as a foundational concept that extends beyond subatomic and quantum scales, finding applications across diverse scientific disciplines. In particle physics, it sheds light on the origin of mass and particle formation [1,2], while in condensed matter physics, it elucidates material behavior and phase transitions [3,4]. In cosmology, symmetry breaking contributes to understanding the distribution of matter and the formation of large-scale structures in the universe [5]. Additionally, in biology, symmetry breaking plays a fundamental role in embryonic development, giving rise to complex and asymmetric structures [6,7,8]. This versatile concept serves as a unifying principle, connecting phenomena from the subatomic to the cosmic and from the quantum to the classical, highlighting its significance in explaining the emergence of patterns, structures, and asymmetry in the natural world.

At subatomic scales, fundamental forces orchestrate a symmetrical dance among particles. However, this balance is disrupted as specific particles gain mass through the Higgs mechanism, marking a subtle yet profound form of symmetry breaking in the subatomic domain [9]. At the molecular scale, the realm of chirality and enantiomerism illustrates symmetry breaking in biomolecular structures. The prevalence of specific handedness in biological molecules, such as amino acids and DNA, underscores the fundamental role of asymmetry in the foundations of life. Unraveling the origin of this chiral bias remains an intriguing puzzle, interwoven with inquiries into the prebiotic conditions that shaped the essential building blocks of life [10].

Ascending to larger scales, the process of crystal formation stands as a macroscopic manifestation of symmetry breaking. As atoms intricately organize into structured arrays, the resulting crystalline structures showcase distinctive symmetries that diverge from the theoretically perfect arrangement dictated by fundamental atomic interactions. This departure accentuates the influence of diverse factors, encompassing temperature gradients, pressure variations, and the presence of impurities, all of which collectively contribute to shaping the ultimate symmetry exhibited by these materials [11].

Transcending the limits of the molecular and atomic domains, the impact of symmetry breaking resonates across astrophysical scales. The very fabric of the universe carries the echoes of symmetry-breaking occurrences during its early stages. With the formation of galaxies, stars, and planets, the once uniform nature of the cosmos transforms into a complex tapestry of structures, eloquently mirroring the indelible imprint left by primordial asymmetries [5].

Delving into the phenomenon of symmetry breaking across diverse scales and scientific domains holds the promise of unraveling fundamental principles that govern our surroundings and shed light on the origins of the cosmos and life itself. Fluctuations and symmetry breaking, deeply embedded since the inception of space and time, emerge as a recurring theme with profound consequences. Central to this exploration is the phenomenon of energy dissipation through entropy production, which serves to illuminate the complexities of symmetry breaking. Our inquiry critically re-examines the interplay between energy dissipation and symmetry breaking across various scales, aiming to unveil profound insights into the emergence of asymmetry in the physical universe. Concentrating on aggregated matter systems, including crystals and self-assembled structures, We examine how fluctuations, nonequilibrium processes, and energy dissipation shape patterns of symmetry breaking.

In this article, we analyze some aspects of symmetry breaking in which, despite expectations of a uniform distribution, a pronounced disproportionality arises in the resulting states. This discrepancy challenges conventional notions and highlights the intricate dynamics at play in diverse systems, ranging from fundamental particle interactions to complex biological processes. While phase changes exemplify scenarios where dynamics provide important insights, our focus is on the energetic and thermodynamic aspects of symmetry breaking. By adopting a mesoscopic nonequilibrium thermodynamics approach, which intrinsically incorporates dynamical processes, we aim to broaden our understanding of symmetry breaking and its implications.

## 2. Symmetry Breaking

Symmetry breaking is a phenomenon in which the symmetry of a system is lost or altered, leading to the emergence of a preferred state or configuration. This can occur spontaneously when the system transitions from a symmetric to a nonsymmetric state without external influence, or it can be induced by external factors, such as temperature changes or external fields [12,13]. Symmetry breaking plays a crucial role in a variety of natural phenomena, including phase transitions, the formation of patterns and structures, and the emergence of complexity in systems ranging from particle physics to biology [14].

Symmetry breaking manifests itself through various mechanisms, including spontaneous, explicit, and fluctuation-induced processes, as discussed in this review [13]. Spontaneous symmetry breaking occurs when the lowest energy state of a system has a symmetry different from that of the equations governing the system. A common example is symmetry breaking in the phase transition of a material, such as the solid-to-liquid phase transition or in the chiral symmetry breaking of organic molecules. When a term in the system description lacks invariance under symmetry-preserving transformations, it leads to explicit symmetry breaking. For example, the application of an external field can break the symmetry that existed in the absence of such a field. Symmetry breaking induced by thermal or quantum fluctuations can amplify small asymmetries in the system, leading to the selection of a particular state.

In particle physics, spontaneous symmetry breaking by the Higgs mechanism elucidates particle mass generation [15]. Quantum systems exhibit explicit symmetry breaking in Bose-Einstein condensates, revealing superfluidity [16]. Active matter systems display fluctuation-induced symmetry breaking, which drives emergent behaviors in self-propelled particles [17]. At the molecular scale, molecular chirality reflects spontaneous symmetry breaking [18]. Crystalline materials undergo explicit symmetry breaking at phase transitions, giving rise to ordered structures [4]. In biology, fluctuation-induced symmetry breaking drives developmental processes, shaping the asymmetry of the organism and determining cell fate [8].

In symmetry-breaking processes, fluctuations emerge as a fundamental aspect shaping the behavior of systems at all scales [19]. At the mesoscale, thermal fluctuations can trigger spontaneous symmetry-breaking events, leading to the formation of ordered structures such as liquid crystals or magnetic domains. Similarly, in biological systems, fluctuations in molecular concentrations or spatial distribution drive symmetry-breaking processes, influencing cell differentiation and developmental patterns [20]. In addition, fluctuations near critical points during phase transitions can amplify small asymmetries, facilitating the selection of preferred states and altering the symmetry of the system [21]. Quantum fluctuations also contribute significantly to symmetry breaking, playing a crucial role in various quantum mechanical phenomena, such as phase transitions and spontaneous symmetry breaking [22].

## 3. Symmetry Breaking across Scales

### 3.1. Subatomic World

Since its inception, the universe has served as a testament to the absence of inherent symmetry. A pivotal moment during the birth of the cosmos witnessed the emergence of consequential asymmetry, tipping the scales in favor of the formation of a slightly greater amount of matter than antimatter. This event has played a profound role in shaping the characteristics of our known universe [1,2]. Known as broken symmetry in particle physics, this phenomenon is well-established at the subatomic level [23]. Yet, a compelling question emerges: Does symmetry breaking confine itself to this minuscule scale? The answer unravels as we delve into the pivotal role of symmetry breaking in comprehending the origins of life, where dissipation assumes a significant role [24] and in which the concepts of symmetry and symmetry breaking first originated in the visible macroscopic systems.

**Figure 1:**
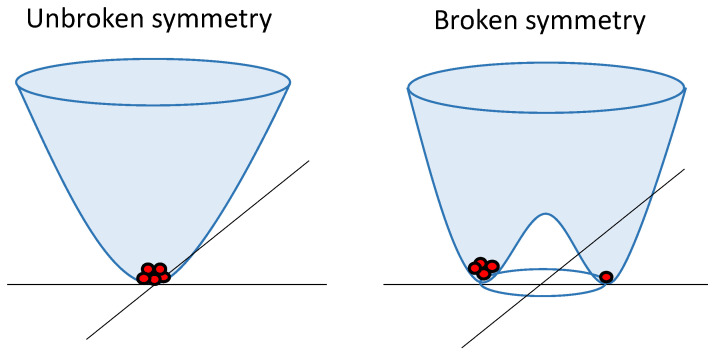
Energylandscapes before and after symmetry breaking: On the left, a symmetric energy landscape where particles (red dots) are uniformly localized in a single state. On the right, an asymmetric landscape follows symmetry breaking, where particles tend to occupy one of the available states.

In quantum field theory, spontaneous symmetry breaking involves the field interacting with itself, resulting in an energy landscape, as depicted in Figure 1. This landscape prompts the field to minimize its energy by rolling downhill. In this process, the field acquires a nonzero strength and randomly (due to the presence of quantum fluctuations) selects a direction in a two-dimensional “phase space”, thereby breaking the initial symmetry. This symmetry breaking gives rise to the emergence of distinct particles, with oscillations within the trough corresponding to massless particles (Goldstone bosons [25]), and oscillations spanning the trough and ascending indicating the existence of massive particles. Note that the proportion of particles in both wells is not equal, indicating a preference for one type of particle, despite both types possessing the same energy. In other words, symmetry is broken, and the reason lies in the process involved, not only in the state’s energy.

In the Higgs mechanism, massless Goldstone bosons combine with other force-carrying particles, imparting mass to them [26]. The Higgs boson becomes a massive particle resulting from jiggling uphill in the energy landscape [9]. Additionally, it is worth noting that Figure 1 has significant importance, as this energy landscape can be observed at various scales and in different processes [27,28], as discussed in the following subsections. It also highlights the possibility of finding more “particles” in one stable state than in the other, even when both states have equal energy.

Within the realm of spontaneous symmetry breaking and the Higgs mechanism, the interplay among quantum fields holds profound implications for the properties of particles. Specifically, the gravitational induction of chirality flips in fermions, notably in the presence of right-handed neutrinos and left-handed antineutrinos, introduces a mechanism for these particles to gain mass upon interacting with Higgs bosons [29]. The effects of perturbatively induced self-gravity, which may be particularly notable for antineutrinos, lead to extended trapping during flight for right-handed antineutrinos compared to their left-handed counterparts, thereby contributing to the asymmetry (disproportion) between matter and antimatter. While this asymmetry is exceedingly subtle, it offers a plausible explanation for the observed prevalence of matter over antimatter, thereby providing insights into the existence of our universe [30]. As initially highlighted by Sakharov [31], an essential prerequisite for baryogenesis, the process that leads to the generation of baryon asymmetry favoring a higher proportion of matter over antimatter in the early universe, is the out-of-equilibrium condition.

### 3.2. Molecular Level

**Figure 2:**
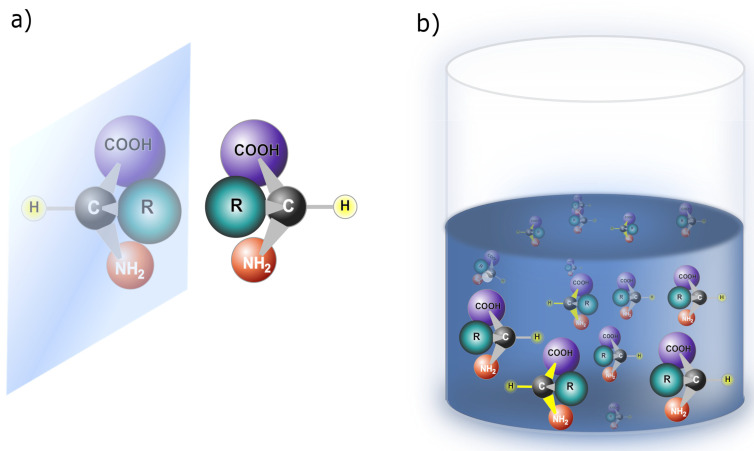
Chiral amino acids: (**a**) Enantiomers: Chiral amino acids are non-super-posable onto their mirror images due to the inability to align the four unique groups on the chiral carbon precisely, even with reorientation in three spatial dimensions [32]. (**b**) Symmetry breaking in a solution of amino acids results in a nonracemic mixture, indicating a disproportion between the two possible enantiomers (chiral amino acids).

**Figure 3:**
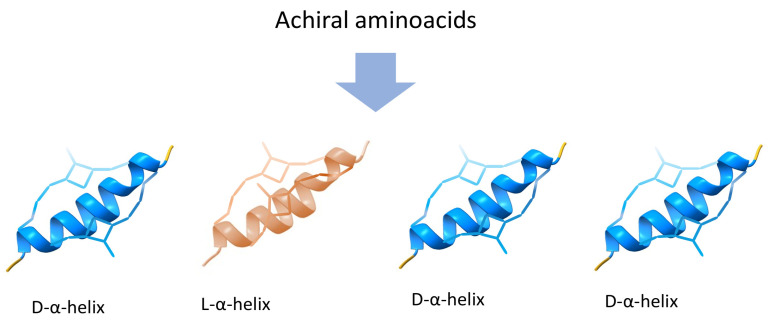
Chiral proteins: Despite being constructed from achiral amino acids, proteins can display mirror images owing to the configuration of the α-helix. Recent studies, such as those by [41], reveal that the population disproportion of protein enantiomers can be controlled.

Symmetry breaking and enantiomers are two interrelated concepts found in molecular and crystalline structures. Symmetry breaking occurs when a system loses or alters its symmetrical properties, leading to an asymmetrical or distorted state [13]. This phenomenon can take place at different scales, affecting the overall structure and properties of the system. Enantiomers, on the other hand, are mirror-image molecules that cannot be superimposed onto each other, as depicted in Figure 2a. The figure illustrates a molecule featuring a stereocenter, specifically a carbon atom, wherein the bonds with functional groups can take two distinct configurations, rendering the molecule chiral [32]. Enantiomers introduce a form of intrinsic asymmetry at the molecular level due to their distinct handedness. Although symmetry breaking can occur in the macroscopic arrangement of molecules or crystals, enantiomers are unique in embodying molecular-level chirality, which results in different physical and chemical properties. In essence, symmetry breaking is a comprehensive concept that encompasses various forms of asymmetry, as seen in the disproportion of enantiomers in a solution (Figure 2b) and the disproportion of chiral proteins (Figure 3). Enantiomers, on the other hand, specifically highlight the unique chiral properties of mirror-image molecules.

At the boundary between chemistry and quantum physics, fundamental symmetry breaking helps us understand how molecular structures and enantiomers emerge [10]. This asymmetry, known as homochirality, is reflected in the prevalence of L-form amino acids in proteins and D-form sugars in DNA/RNA. However, the reasons behind this inherent disparity are still an open question, leading researchers to explore the factors that trigger symmetry breaking. At equilibrium, the free energy change $\Delta_{r}G$ required for the formation of possible enantiomers is practically the same, resulting in weak or no chiral symmetry breaking. However, experimental evidence shows that forces such as polarized light, shear, temperature gradients, and others can either hinder the formation or enhance the decomposition of one enantiomer over the other [33,34,35,36,37,38]. This observation is of particular significance in the origin of life, especially considering the likely extreme and nonequilibrium environments during prebiotic conditions [39,40].

Under local equilibrium [42,43] or local quasi-equilibrium [44], the free energy changes consist of reversible and irreversible contributions:

(1) $$\Delta G=\Delta_{r}G+\Delta_{i}G$$

The reversible free energy change depends on the state variables, whereas the irreversible free energy change, $\Delta_{i}G=-T\Sigma$, is given through the total entropy produced Σ in the process, defined as follows:

(2) $$\Sigma =\int_{0}^{\infty }\sigma \left(t\right)dt$$

where σ is the entropy production rate. At the molecular scale, energy-release processes primarily involve the formation and breaking of chemical bonds and hydrogen bonds. These events may be driven by differences in free energy between the initial and final states. Additionally, external factors like temperature gradients or stress can influence chemical reactions and phase transitions. At this level, we can quantify the probability ρ(γ,t) of observing the system in a specific configuration $\gamma =[\gamma_{1},\ldots ,\gamma_{i},\ldots ,\gamma_{n}]$ at time *t* [43], where $\gamma_{i}$ represents an internal coordinate associated with the *i*-th process. The conservation of probability is elucidated by the conservation law

(3) $$\frac{\partial \rho }{\partial t}+v\cdot \nabla_{\gamma }\rho =-\nabla_{\gamma }\cdot J$$

in which J is the probability current associated with the *n* processes taking place in the system, v the fluid velocity field, and $\nabla_{\gamma }$ is the gradient operator along γ-space.

On the other hand, in a system in which there is momentum and heat transport, the entropy production rate can be expressed as [42,43,45]

(4) $$\sigma \left(t\right)=-\frac{1}{T}\int_{\gamma }\int_{r}\left(J\cdot \nabla_{\gamma }\mu +J_{q}\cdot \nabla_{r}T+\Pi :\nabla_{r}v\right)d\gamma dr$$

where $J_{q}$ is the heat flux, Π the viscous pressure tensor, and $\nabla_{r}$ the gradient operator in the Euclidean space.

The expression for the current of the i-th process then results from the linear coupling with the conjugate thermodynamic forces:

(5) $$J_{i}=-\frac{L_{ii}}{T}\frac{\partial \mu }{\partial \gamma_{i}}-\frac{1}{T}{\underline{L}}_{qi}\cdot \nabla_{r}T-\frac{1}{T}{\underline{\underline{L}}}_{vi}:\nabla_{r}v$$

in which we have made the assumption that the process occurs on a surface, allowing the coupling of processes that originally exhibit different tensorial orders. The coefficients $L_{ii}$,${\underline{L}}_{qi}$, and ${\underline{\underline{L}}}_{vi}$ form the Onsager coefficients’ matrix.

It was shown in [43] that by assuming local equilibrium in the space of the reaction coordinate of an activated process and performing a coarse graining, one obtains a nonlinear expression of the activation current in terms of the affinity A=Δμ (chemical potential difference between initial and final states), which corresponds to the law of mass action. This mesoscopic nonequilibrium thermodynamics approach constitutes a generalization of classical nonequilibrium thermodynamics, offering a general framework under which activated processes, which are intrinsically nonlinear, can be studied. Explicitly, we have

(6) $$J_{i}=-\frac{D_{i}\rho }{k_{B}T}\frac{\partial \mu }{\partial \gamma_{i}}=-D_{i}\exp-\phi /k_{B}T\frac{\partial z}{\partial \gamma_{i}}$$

where we defined the diffusivity along the internal coordinate $\gamma_{i}$ as $D_{i}=L_{ii}/k_{B}$, the fugacity as $z=exp(\mu /k_{B}T)$, and the chemical potential as $\mu =k_{B}T\ln\rho +\phi \left(\gamma_{i}\right)$ with ϕ as an energy barrier in $\gamma_{i}$. Coarse graining the description by integrating in gamma-space ($\gamma_{i}\in \left[0,1\right]$), one obtains the law of mass action:

(7) $$\langle J_{i}\rangle =\int_{0}^{1}J_{i}d\gamma_{i}=-D_{0}\left(z\left(1\right)-z\left(0\right)\right)=k_{0}\left(1-\exp\left(A/k_{B}T\right)\right)$$

in which $D_{0}=D_{i}\int_{0}^{1}\exp\left(\phi /k_{B}T\right)d\gamma_{i}$ and $k_{0}=D_{0}\exp\left(-\mu \left(0\right)/k_{B}T\right)$. One then concludes that the activation current is linear in the fugacity differences but nonlinear in the affinity.

### 3.3. Enantiomeric Crystals

Enantiomeric crystals, highlighting the profound influence of molecular chirality on crystal symmetry, are encountered across diverse scientific disciplines. A striking illustration is evident in the pharmaceutical field, where enantiomeric crystals of drug compounds can exhibit markedly distinct pharmacological activities [46]. The thalidomide case serves as a noteworthy example, where one enantiomer led to severe birth defects, while the other demonstrated safe sedative properties [47]. Enantiomeric crystals also find applications in materials science due to their unique optical properties. The chirality of mesocrystals has recently emerged as a prominent and dynamic topic in nanoresearch. The significant exploration and study of the unique and intriguing properties of chiral mesocrystals underscore their relevance in both fundamental research and potential applications [48,49,50].

The diverse proportions of enantiomeric crystals can be understood by applying thermodynamic principles [51]. To illustrate this, it is essential to calculate the free energy change in the process. This computation is crucial for revealing the probability linked to the observation of a specific structural configuration

(8) $$p\propto \exp\left(-\frac{\Delta G}{k_{B}T}\right)$$

In scenarios where only two potential enantiomers are involved, the deviation from the equilibrium distribution can be characterized by χ, defined as χ=2p−1. From this parameter, we can calculate the enantiomeric excess percentage

(9) %e.e.=χ∗100%

**Figure 4:**
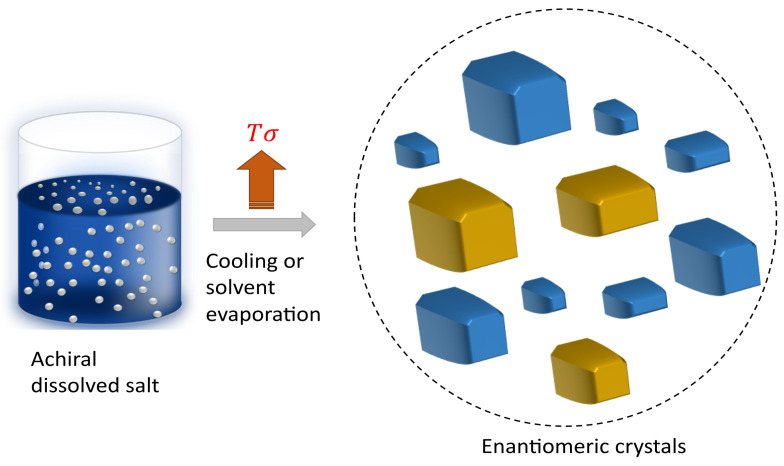
Enantiomeric crystals and symmetry breaking: The genesis of L- and D-crystals (depicted by blue and yellow cubes), in different ratios, originates from achiral compounds (illustrated as white dots). Symmetry breaking occurs as driving forces, namely temperature gradients and local oversaturation, emerge during the system’s cooling or the solvent’s evaporation, leading to irreversible energy dissipation Tσ.

The D and L enantiomeric configurations of chiral crystals exhibit specular macro- and micromorphologies and possess opposite directions of optical rotation. Secondary nucleation and Viedma ripening are two well-known and complementary mechanisms that explain chiral symmetry breaking [34,52,53]. In addition, the presence of an enantiomeric excess has also been attributed to differences in activation energies between the enantiomers, leading to different rates of formation [40,54]. These variations in the nucleation stage, proposed by both secondary nucleation and Viedma ripening mechanisms, are influenced by shear rates affecting crystal and nuclei formation, which could be related to variations in activation energies during the nucleation stage. Therefore, nonequilibrium conditions during crystallization play a crucial role [55,56,57], determining the symmetry-breaking phenomenon and not only the initial conditions, as illustrated in Figure 4. In this context, the final states (enantiomeric crystals) possess identical energy levels, yet they are present in varying proportions. This disparity underlines a symmetry breaking, attributed to external conditions that influence energy dissipation during the crystallization process and consequently affect the activation energy.

Experiments consisting of evaporating the solvent to obtain NaClO_3_ crystals [34], performed by stirring the sample, revealed a significant disproportion in the concentrations of both enantiomeric crystals of NaClO_3_. In a recent study, it was found that the energy dissipation rate of the phase change $T\sigma_{ph}$ and heat transfer $T\sigma_{T}$ determines the enantiomeric excess percentage in the case of enantiomeric crystals of NaClO_3_ [51]:

(10) $$T\sigma_{ph}\left(y,t\right)=-\left({\dot{r}}_{L}\Delta z_{L}+{\dot{r}}_{D}\Delta z_{D}\right)$$

(11) $$T\sigma_{T}\left(y,t\right)=\frac{\kappa }{T}{\left(\frac{\partial T}{\partial y}\right)}^{2}$$

${\dot{r}}_{L}$ and ${\dot{r}}_{D}$ are the nucleation rates of both enantiomers, whereas $\Delta z_{L}$ and $\Delta z_{D}$ are the driving force (fugacity difference between liquid and solid state). Moreover, κ is the thermal conductivity of the system and *T* is the temperature. In this case, the activation energy $E_{a}$ in the formation of the nuclei differs between enantiomers, and the difference ($\Delta E_{a}$) is proportional to the energy dissipated per salt mole in the process, i.e., $\Delta E_{a}=\Delta_{i}G=T\Sigma$.

**Figure 5:**
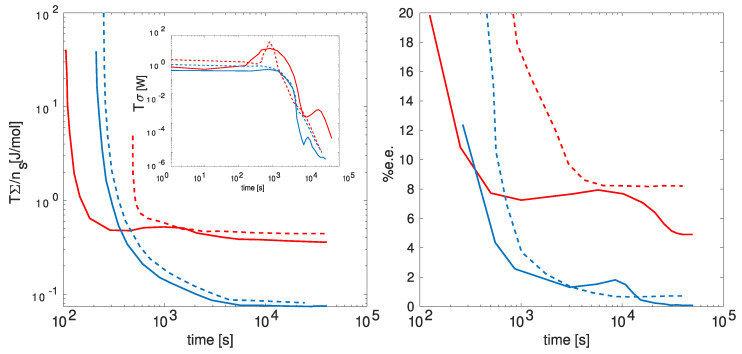
Nonequilibrium thermodynamic data on symmetry breaking [51]: Formation of enantiomeric crystals under varied cooling conditions and salt concentrations. The left plot illustrates the total energy dissipated per mol of solid salt over time, with the inset depicting the energy dissipation rate. The right plot shows the enantiomeric excess as a function of time. Red lines represent high salt concentration, while blue lines denote the lowest salt concentration permitting crystal formation. Dotted lines signify enhanced cooling, while continuous lines depict natural (slower) cooling.

In Figure 5, the anticipated behavior of the energy dissipation rate (Tσ), energy dissipated per solid salt mol ($T\Sigma /n_{s}$), and the enantiomeric excess percentage (%e.e.) is presented over time. The figure delineates these behaviors under distinct cooling conditions (depicted by red and blue lines) and various initial concentrations of dissolved salt (continuous and dashed lines). These specified conditions exert influence over the energy dissipation, consequently impacting the symmetry breaking observed in the enantiomeric excess. Notice that as the energy dissipation reaches higher peaks, there is a corresponding increase in the energy dissipated per mole of salt, leading to a higher enantiomeric excess.

### 3.4. Mesoscopic Domain

The concept of symmetry breaking is responsible for creating homochirality, a phenomenon that is present across different levels of existence and fields. The explanation for the emergence of biological chirality can be found in the homochirality of chemical and physical systems. The directed and selective self-assembly of components, combined with the dissipation of energy and autocatalytic networks, drives self-organization [18,58,59,60,61,62]. This intersection of physical chemistry and biology witnesses the spontaneous emergence of self-assembled and self-organized structures driven by the dissipation of energy [28,59,60,63]. Lately, there has been a lot of thought given to whether the dissipation of energy can help explain why symmetry is broken. The focus of this inquiry is to better understand how homochiral structures (enantiomers) come to be, as they are the precursors to self-organized structures.

Before moving on to discussions of self-assembled and enantiomeric structures, we discuss the dynamical domain of spatiotemporal patterns and phase changes in dissipative systems as examples of symmetry breaking [14]. Comprehensive reviews illustrate the various patterns observed in hydrodynamic systems, nonlinear optics, and biological media [20]. The interplay between deterministic chaos and spatiotemporal chaos underscores the complexity of these systems and provides insights into symmetry-breaking behaviors at the mesoscopic scale. Further exploration of symmetry-breaking phenomena integrates concepts of bifurcation, Landau phase transition theory, and rare emergent events [19]. Within this mesoscopic context, fast dynamics manifest as rapidly varying stochastic processes, while mid-level dynamics exhibit nonlinearity, leading to jump-like transitions between wells, synonymous with dynamical symmetry breaking. This understanding lays the foundation for exploring the formation of self-assembled structures and enantiomeric phenomena within dissipative systems.

**Figure 6:**
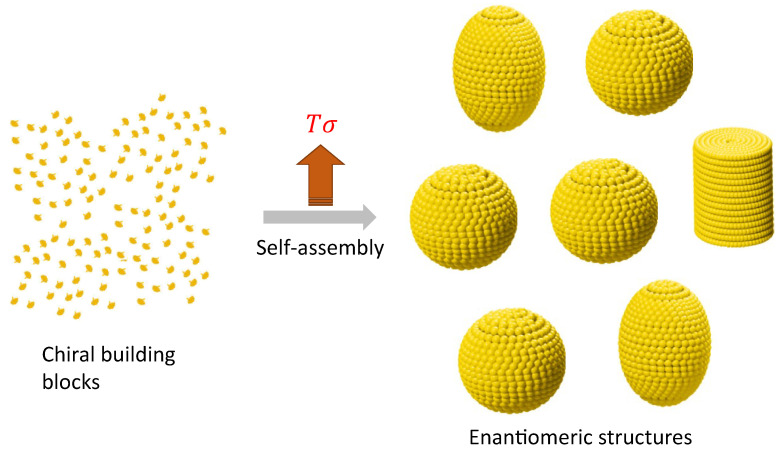
Polymorphic mesostructures derived from self-assembled chiral building blocks: Symmetry is disrupted as equiprobable mesostructures, possessing identical energy, emerge in varying proportions due to dissipative-driven self-assembly processes. It is noteworthy that the self-assembly can yield more than one polymorph, representing distinct enantiomeric structures.

Enantiomeric structures can be elucidated as formations stemming from nonequilibrium self-assembly (NESA) processes, where the fundamental components consist of chiral molecules or chiral building blocks, as illustrated in Figure 6. A conceptual framework for comprehending out-of-equilibrium crystallization through nonequilibrium self-assembly (NESA) processes has been previously proposed [64]. At this level, enantiomers can be more than two, also termed polymorphs. In a steady state, a system with more than two enantiomers can emerge due to the interplay of self-assembly and symmetry breaking. The probability for the η configuration is [28]

(12) $$p\left(\eta \right)=p\left(\eta_{0}\right)e^{-\beta \Delta G(\eta )}$$

**Figure 7:**
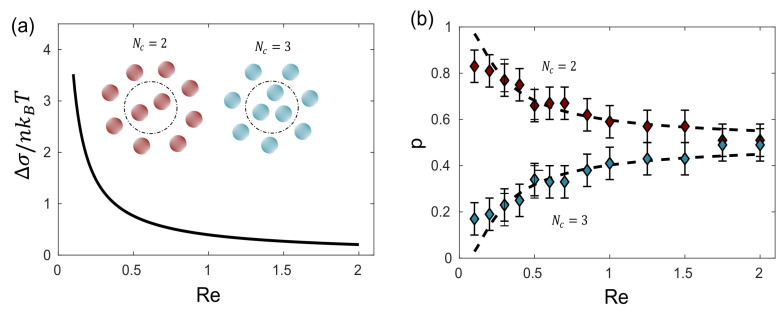
Magnetohydrodynamic self-assembled polymorphs: The structure manifests two potential configurations, $N_{c}=2$ and $N_{c}=3$, distinguished by the number of central particles. The (**a**) illustrates the difference in entropy production rate between both polymorphs (inset, $N_{c}=2$ and $N_{c}=3$) as a function of the Reynolds number, while (**b**) showcases the probability of observing both polymorphs in relation to the Reynolds number. Experimental results are denoted by dots, and dashed lines represent the estimated probability derived from theory, considering energy dissipation as the primary variable.

In the case of the steady-state magnetohydrodynamic self-assembly, two enantiomers (polymorphs) can arise. In this case, a set of small magnetic particles suspended on a liquid–air interface under the influence of a magnetic field generated by a rotating magnet [65], which can be controlled to contain different Reynolds numbers. The movement of the particles perturbs the fluid’s state of rest giving rise to the formation of a hydrodynamic pattern, which in turn induces the assembly of the particles that orbit around the axis of the magnet (inset Figure 7). The energy supplied by the rotating magnet is dissipated in the fluid due to its viscous nature. The entropy production for a configuration $N_{c}$ is given by

(13) $$\sigma \left(N_{c}\right)=-\frac{1}{T}\int v:\Pi dr$$

where the velocity field v and the tensor Pi depend on the particle configuration in the system $N_{c}$. It was proven that the probability of observation is given by the entropy production rate:

(14) $$\frac{p(N_{c}=2)}{p(N_{c}=3)}=\exp\left(-\beta \left(T\Delta \sigma \right)\right)$$

in which σ is the entropy production rate per molecule of moving fluid around the particles and $\Delta \sigma =\sigma \left(N_{c}=2\right)-\sigma \left(N_{c}=3\right)$.

### 3.5. Living Organisms

Living organisms are complex, self-organized structures [60] composed of chiral molecules, entities that inherently exhibit asymmetry [66,67].

The simplicity of left-right asymmetry offers a basis for meaningful comparisons across different organisms. Exploring how this asymmetry varies, is inherited, and operates at the molecular level provides surprising insights into the evolution of development [68,69]. Firstly, this type of asymmetry often arises from noninherited traits as much as from genetic mutations, suggesting a reciprocal relationship where traits can influence genes over time. Conditions and diet [70], along with dissipation, as seen in studied self-organized structures [60], may play crucial roles. Secondly, the development of left-sided preference in hearts varies widely among animals. This developmental process might have been borrowed from an older asymmetrical organ system in chordates [70]. Hence, external conditions, initial chiral or asymmetric structures, genes, and mutations collectively influence the likelihood of encountering, for instance, a left-sided heart. This raises the question: Can we analyze symmetry breaking in organisms by studying dissipation under the usual conditions they face? This mirrors the approach taken in understanding cancer evolution, where carcinogenic tissues are viewed as self-organized structures [71].

Handedness, one of the most extensively studied asymmetries in human motor behavior, refers to the preference for using one hand over the other. This inclination is universally observed and consistent among Homo sapiens. Remarkably, across all human cultures, approximately 90% of individuals favor their right hand, while the remaining 10% exhibit a preference for their left hand [72]. Notably, other socially intelligent mammals do not display such a consistent handedness pattern at the species or population level. This lateralization in hand use is indicative of underlying asymmetry in cerebral structure, and the evolution of handedness appears to be linked to other lateralized abilities such as footedness, earedness, and eyedness. This intricate interplay of brain, hand, and speech forms an adaptive complex that is characteristic of our species [73,74].

Various theories, including genetic, environmental, social, and behavioral perspectives, have been proposed to comprehend the symmetry breaking observed in the dominant hand; however, consensus remains elusive in the literature [75]. A compelling question arises: Can we integrate these diverse theories within the realm of the mind and consciousness, especially considering that energy emerges as a common variable? The processes unfolding—from individual neuronal interactions and biochemical reactions facilitating thoughts or cascades of thoughts to the intelligence enabling communication and communal life—undoubtedly involve changes in energy and energy flows. Could future theories explore the notion that more efficient energy flows or considerations of energy dissipation, the loss of energy, and information might shed light on understanding handedness, similar to phenomena observed at the molecular and mesoscopic scales?

## 4. Discussion and Conclusions

The exploration of symmetry-breaking and its connection to energy dissipation traverses various scales, providing profound insights into the fundamental principles governing a myriad of phenomena. Starting from the cosmic scale, the birth of the universe marked a crucial asymmetry, resulting in the dominance of matter over antimatter. This broken symmetry, firmly established in particle physics at the subatomic level, continues to exert its influence on the emergence of life as we know it, particularly evident in the homochirality of biomolecules.

A pivotal proposition has arisen regarding the significance of energy dissipation in elucidating symmetry breaking. The dissipation of energy through entropy production has emerged as a consistent and compelling phenomenon, influencing the system’s free energy and leading to the establishment of an effective free energy potential. This concept underwent thorough exploration across diverse scales, ranging from elemental particles to aggregated matter systems like crystals.

The inquiry extended to human-scale observations, unveiling an inherent connection between our perception of asymmetry and our chiral nature. Biological chirality, manifest in cellular processes and psychological aspects, underwent scrutiny in the context of self-assembly, autocatalytic networks, and energy dissipation at the intersection of physical chemistry and biology.

Symmetry breaking has proven instrumental in crystallography, shedding light on how external conditions and nonequilibrium processes govern deviations from perfect arrangements and symmetric properties during crystal formation. Enantiomeric crystals, formed through dissipation-driven symmetry breaking, vividly illustrate the transformation from achiral compounds to distinct L- and D-crystals.

The discussion converged on the overarching theme of exploring symmetry breaking across scales, with a particular focus on aggregated matter systems such as crystals and self-assembled structures. The intricate interplay of fluctuations, nonequilibrium processes, and energy dissipation emerged as pivotal factors shaping the patterns of symmetry-breaking.

In essence, this discussion delves into the coherence of symmetry-breaking phenomena across different scales and disciplines, revealing a common thread in the dissipation of energy. From the cosmic to the molecular scale, the exploration of this relationship provides valuable insights into the fundamental principles governing the emergence of asymmetry in the physical universe.
